# Lyophilized Polyvinyl Alcohol and Chitosan Scaffolds Pre-Loaded with Silicon Dioxide Nanoparticles for Tissue Regeneration

**DOI:** 10.3390/molecules29163850

**Published:** 2024-08-14

**Authors:** Andrés Felipe Niebles Navas, Daniela G. Araujo-Rodríguez, Carlos-Humberto Valencia-Llano, Daniel Insuasty, Johannes Delgado-Ospina, Diana Paola Navia-Porras, Paula A. Zapata, Alberto Albis, Carlos David Grande-Tovar

**Affiliations:** 1 Grupo de Investigación de Fotoquímica y Fotobiología, Universidad del Atlántico, Carrera 30 Número 8-49, Puerto Colombia 081008, Colombia; afniebles@mail.uniatlantico.edu.co (A.F.N.N.); dgaraujo@mail.uniatlantico.edu.co (D.G.A.-R.); 2 Grupo Biomateriales Dentales, Escuela de Odontología, Universidad del Valle, Calle 4B Número 36-00, Cali 760001, Colombia; carlos.humberto.valencia@correounivalle.edu.co; 3 Departamento de Química y Biología, División de Ciencias Básicas, Universidad del Norte, Km 5 Vía Puerto Colombia, Barranquilla 081007, Colombia; insuastyd@uninorte.edu.co; 4 Grupo de Investigación Biotecnología, Facultad de Ingeniería, Universidad de San Buenaventura Cali, Carrera 122 Número 6-65, Cali 760001, Colombia; jdelgado1@usbcali.edu.co (J.D.-O.); dpnavia@usbcali.edu.co (D.P.N.-P.); 5 Grupo de Polímeros, Facultad de Química y Biología, Universidad de Santiago de Chile (USACH), Santiago 9170020, Chile; paula.zapata@usach.cl; 6 Grupo de Investigación en Bioprocesos, Facultad de Ingeniería, Universidad del Atlántico, Carrera 30 Número 8-49, Puerto Colombia 081008, Colombia; albertoal-bis@uniatlantico.edu.co

**Keywords:** chitosan, lyophilized scaffolds, polyvinyl alcohol, silicon dioxide nanoparticles

## Abstract

Materials with a soft tissue regenerative capacity can be produced using biopolymer scaffolds and nanomaterials, which allow injured tissue to recover without any side effects or limitations. Four formulations were prepared using polyvinyl alcohol (PVA) and chitosan (CS), with silicon dioxide nanoparticles (NPs-SiO_2_) incorporated using the freeze-drying method at a temperature of −50 °C. TGA and DSC showed no change in thermal degradation, with glass transition temperatures around 74 °C and 77 °C. The interactions between the hydroxyl groups of PVA and CS remained stable. Scanning electron microscopy (SEM) indicated that the incorporation of NPs-SiO_2_ complemented the freeze-drying process, enabling the dispersion of the components on the polymeric matrix and obtaining structures with a small pore size (between 30 and 60 μm) and large pores (between 100 and 160 μm). The antimicrobial capacity analysis of Gram-positive and Gram-negative bacteria revealed that the scaffolds inhibited around 99% of *K. pneumoniae*, *E. cloacae*, and *S. aureus* ATCC 55804. The subdermal implantation analysis demonstrated tissue growth and proliferation, with good biocompatibility, promoting the healing process for tissue restoration through the simultaneous degradation and formation of type I collagen fibers. All the results presented expand the boundaries in tissue engineering and regenerative medicine by highlighting the crucial role of nanoparticles in optimizing scaffold properties.

## 1. Introduction

Tissue injuries are prevalent in humans and represent a significant global public health challenge due to their complexity and the lengthy recovery processes associated with conventional treatments. Various factors, such as genetic disorders, trauma, accidental injury, and severe disease, can cause extensive tissue damage, hindering effective regeneration [1]. Treatments, such as tissue transplants (autografts or allografts), are often insufficient for skin wounds due to long recovery times, the risk of bacterial infections, and the possibility of tissue necrosis, resulting in inflammation and pain for the patient [2]. In response to these challenges, the scientific community has explored innovative methods that promote the effective regeneration of injured tissue [3].

Tissue engineering emerges as a promising solution, using biomaterial-based scaffolds to facilitate cell adhesion and proliferation, promoting the long-term recovery of tissue architecture and facilitating the exchange of nutrients and energy essential for cell proliferation in skin tissue [4]. However, the efficacy of these scaffolds is highly dependent on their morphological, biodegradable, and biocompatible properties, which should avoid cytotoxic or immunogenic effects [5]. In addition, scaffold materials must be resorbable to stimulate and accelerate the tissue-healing process [6]. In this context, polyvinyl alcohol (PVA) and chitosan (CS) have proven to be ideal materials for scaffold fabrication due to their unique properties. 

PVA is a water-soluble synthetic polymer with a high capacity to store electrochemical charge and is known for its biocompatibility and biodegradability [7]. These characteristics have made PVA widely used in biomedical applications, particularly in the formation of scaffolds together with other polymers such as chitosan, collagen, and alginate [8,9]. Recently, the use of PVA in tissue regeneration has been intensively investigated. For example, Scott et al. [10] explored carbon nanotube-enhanced PVA scaffolds for improved conductivity and cell support in cardiac tissue-engineering applications [10]. In addition, Aldakheel et al. developed [11] PVA and CS matrices that showed significant improvements in wound healing and antimicrobial activity by combining these materials with silver nanoparticles [11]. Recent studies have also demonstrated the potential of PVA in sustainable and biomedical applications. For instance, a study on Aloe vera gel-reinforced biodegradable starch-PVA blends showed improved mechanical and barrier properties of the material for sustainable packaging of green chilies [12]. Similarly, the extraction of cellulose from green algae *Ulva ohnoi* and its application in PVA-based antibacterial composite films incorporated with zinc oxide nanoparticles have highlighted their antimicrobial capacity and potential in biomedical applications [13]. Additionally, the fabrication of an antimicrobial colorimetric pad for meat packaging, based on PVA aerogel with the incorporation of anthocyanins and silver nanoparticles, demonstrated the effective detection of meat quality, enhancing food safety [14].

Chitosan (CS) is a natural polysaccharide derived from the deacetylation of chitin and is known for its biocompatibility, biodegradability, and low toxicity [15]. Its solubility in acidic aqueous media makes it a versatile material for medical applications, being recognized by the FDA as safe for consumption (GRAS) [16]. Murphy et al. demonstrated that chitosan scaffolds can promote effective bone regeneration when combined with hydroxyapatite [17], while Xing et al. investigated the ability of chitosan membranes to facilitate chronic wound healing through controlled drug release [18]. PVA and CS blends are valued for the production of biodegradable polymers with mechanical properties suitable for biomedical applications [19]. However, these blends face challenges due to the high water solubility of PVA, which can lead to the embrittlement of the polymeric membranes [20] and high catalytic activity. SiO_2_ has numerous biotechnological applications in the enzyme, agricultural, and textile industries [21]. Hydrogen-bonding interactions between PVA and CS facilitate rapid solidification of the blend, resulting in an inhomogeneous polymeric structure [22]. 

To overcome these limitations, the incorporation of nanocomposites, such as inorganic nanoparticles, has been investigated to improve the mechanical properties by decreasing the glass transition temperature (Tg) and increasing the ionic conductivity of the polymer blend [23]. Mainly, silicon dioxide nanoparticles (NPs-SiO_2_) exhibit outstanding properties such as semi-conductivity, absorption of a wide range of radiations, and high catalytic activity [24]. These nanoparticles have numerous biotechnological and clinical applications, including their use in multifunctional platforms for diagnostics and gene therapy [25]. In addition, SiO_2_ is biocompatible and exhibits low toxicity, making it ideal for medical applications [26]. NPs-SiO_2_ can stimulate the development of fibroblasts and endothelial cells, significantly improving the wound-healing process due to its biocompatibility, thermal stability, and optical properties [26]. 

Comparing the findings of recent studies on the antibacterial properties of chitosan-based nanoparticles, it is observed that these combinations have shown considerable effectiveness against various bacterial strains. For instance, studies have demonstrated that chitosan composites with silver nanoparticles exhibit notable antimicrobial activity, inhibiting the growth of bacteria such as *Escherichia coli* and *Staphylococcus aureus*. This antimicrobial effect is likely due to the silver nanoparticles disrupting bacterial cell walls and interfering with cellular processes [27]. Similarly, ZnO nanoparticles incorporated into chitosan films have shown a significant reduction in bacterial viability, attributed to the generation of reactive oxygen species by ZnO nanoparticles, which damage bacterial cell membranes and DNA [28].

This study uses the freeze-drying method to integrate silica nanoparticles into a PVA and CS matrix. The addition of silica nanoparticles not only improves the mechanical and thermal properties of the scaffold but also enhances its antimicrobial capacity and biocompatibility, aspects that have not been widely explored in previous research. The freeze-drying method creates a uniform porous structure that facilitates the retention and homogeneous distribution of nanoparticles within the polymer matrix, which is crucial to maintaining the stability of nanoparticles in matrices with large pores [29]. Unlike previous studies, this research not only focuses on the physicochemical and antimicrobial properties of the scaffolds but also includes a detailed evaluation of their biocompatibility and long-term stability through in vivo studies in biomodels, providing a comprehensive view of their potential in clinical tissue-regeneration applications.

## 2. Results and Discussion

### 2.1. Characterization of Silicon Dioxide Nanoparticles (NPs-SiO_2_)

#### 2.1.1. FTIR Analysis NPs-SiO_2_

**Figure 1:**
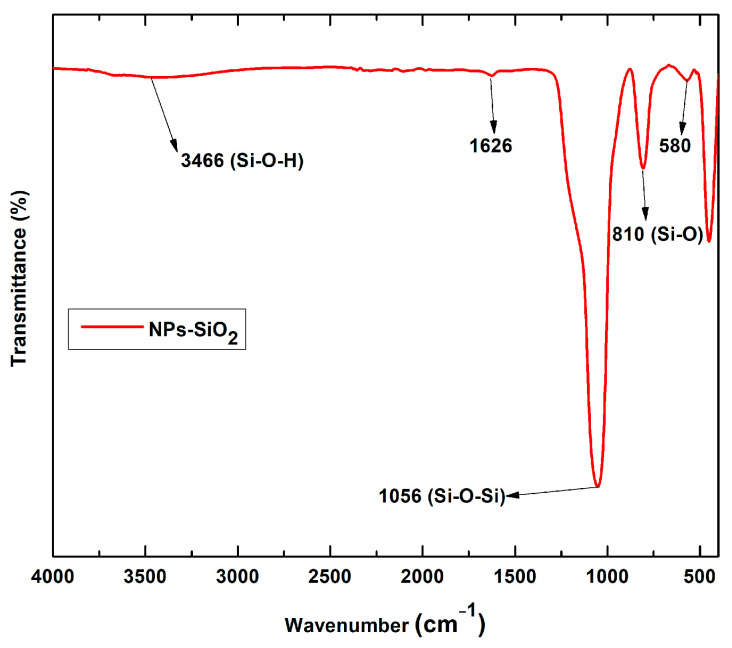
FT-IR spectra of SiO_2_ nanoparticles.

Figure 1 shows the FT-IR spectra of pristine SiO_2_-NPs. The band at 3466 cm^−1^ is due to the characteristic O-H absorption belonging to the symmetric stretching of silanol and 1626 cm^−1^ scissors’ bending vibration of molecular water [30]. Silicon dioxide nanoparticles showed typical absorption bands at 810 cm^−1^ and 1056 cm^−1^ due to siloxane bonding and silanol symmetric bending and stretching. These bands are a superposition of several SiO_2_ peaks due to residual organic groups [31,32].

#### 2.1.2. XRD Analysis NPs-SiO_2_

**Figure 2:**
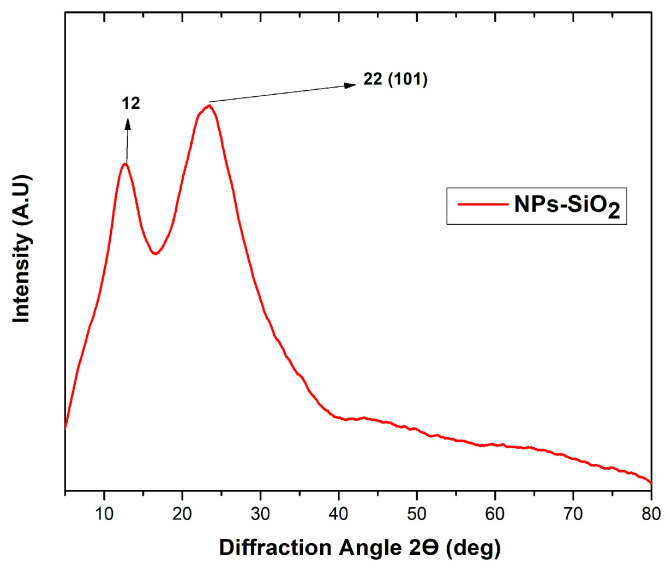
XRD analysis of SiO_2_ nanoparticles.

The X-ray diffraction technique investigated silicon dioxide nanoparticles’ amorphous and crystalline nature. In the analysis, the amorphous nature of the NPs-SiO_2_ was confirmed by the base-catalyzed reaction in the sol-gel method, as evidenced in Figure 2. The broad peak observed at 22° coincides with the central peak of the (101) plane of crystalline silicon oxide, a typical form of amorphous behavior of the nanoparticles [30]. In addition, a secondary peak was observed at 12°, suggesting internal stresses in the material. As reported in other investigations, the base- or acid-catalyzed reaction shows amorphous peaks. In addition, the absence of any ordered crystalline structure is indicated, which may be due to a decreased particle size or mechanical stress in the structure [30]. This structural characterization provides a detailed understanding of the nature of the NPs-SiO_2_. Regarding their toxicity, it was determined that NPs-SiO_2_ in the amorphous state exhibit low toxicity and prevent harmful effects on cells [33].

#### 2.1.3. TEM Analysis NPs-SiO_2_

**Figure 3:**
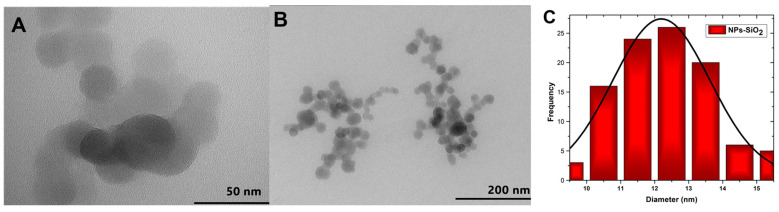
TEM of SiO_2_ nanoparticles, (**A**) 50 nm; (**B**) 200 nm; (**C**) Particle size histogram.

The morphology of the silicon dioxide nanoparticles was analyzed using the TEM technique, as shown in Figure 3A. The spherical nature of the NPs-SiO_2_ can be evidenced since the sol-gel synthesis method presents multiple hydrolysis, polycondensation, and polymerization reactions generating spherical particles of high purity and homogeneity [34]. Clusters of different shapes and sizes can also be observed (Figure 3B). This phenomenon may be due to electrostatic attraction or Van der Walls forces between the particles [35]. Quantitative analysis of the silicon dioxide nanoparticles was performed using ImageJ bundled with 64-bit Java 8 software (Figure 3C). About 100 nanoparticles with an average size of 12.19 ± 0.13 nm were measured. 

### 2.2. Characterization of PVA/CS/NPs-SiO_2_ Scaffolds

#### 2.2.1. FT-IR Analysis of PVA/CS/NPs-SiO_2_ Scaffolds

**Figure 4:**
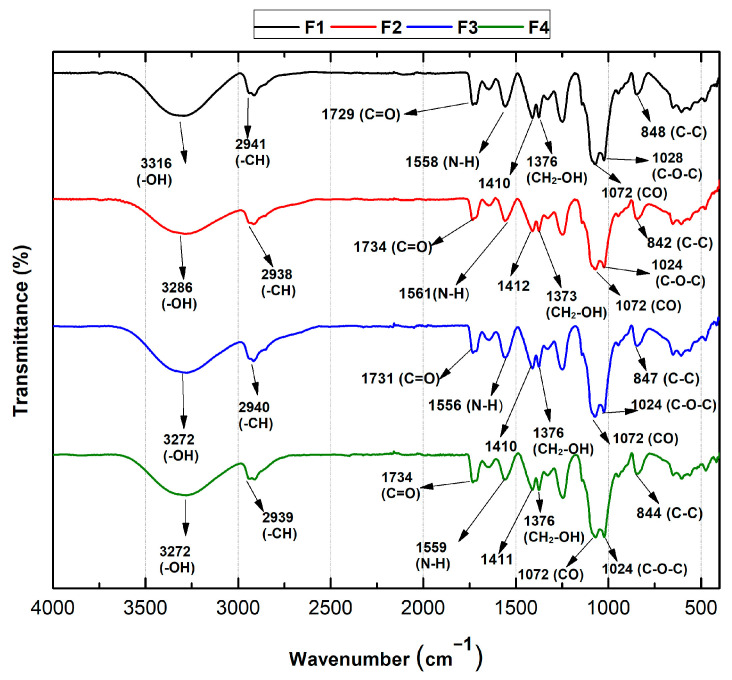
FT-IR spectra of PVA/CS/SiO_2_-NPs scaffolds. F1, 70%PVA/30%CS; F2, 70%PVA/29.5%CS/0.5%NPs-SiO_2_; F3, 70%PVA/29%CS/1%NPs-SiO_2_; F4, 70%PVA/28%CS/2%NPs-SiO_2_.

As shown in Figure 4, Fourier transform infrared spectroscopy (FT-IR) was used to verify the functional groups of the PVA/CS/NPs-SiO_2_ scaffolds.

In general, the formulations based on chitosan and polyvinyl alcohol presented good compatibility along with the incorporation of the NPs-SiO_2_. For all formulations, vibrational modes between 1024–1028 cm^−1^ and 842–848 cm^−1^ belonging to C-O-C and C-C, respectively, are presented, which are found to be related to characteristic chitosan crystallization peaks confirmed in XRD. This is due to the hydrogen bonds between the hydroxyl groups of PVA and the amino and hydroxyl groups of chitosan [36]. However, for F2, the intensity of the bands decreased and showed a slight shift, probably due to the interaction with the NPs-SiO_2_ and the polymeric mixture. Likewise, a band is observed at 1072 cm^−1^ that reflects the stretches corresponding to the C-O bonds attributed to the monomeric units of PVA [37]. Similarly, for all formulations, the amino (N-H) band was observed between 1556 and 1561 cm^−1^ due to symmetrical NH_3_^+^ deformations resulting from the ionization of primary amino groups of chitosan in the acidic medium. In addition, the bands between 1734 and 1729 cm^−1^ corresponding to the stretching of the C=O bonds of the acetate group of PVA were observed. Asymmetric strain bands are also evident for the CH-CH_2_ alkyl groups between 2938 and 2941 cm^−1^ [38]. On the other hand, a band is present at 3316 cm^−1^ corresponding to the -OH group in F1, which shifts to 3272 cm^−1^ for the F3 and F4 formulations. This shift is attributed to the superposition of the hydroxyl groups of PVA with the amino and hydroxyl groups of chitosan, suggesting the formation of many hydrogen bonds between PVA and chitosan [38]. 

#### 2.2.2. XRD Analysis of PVA/CS/NPs-SiO_2_

**Figure 5:**
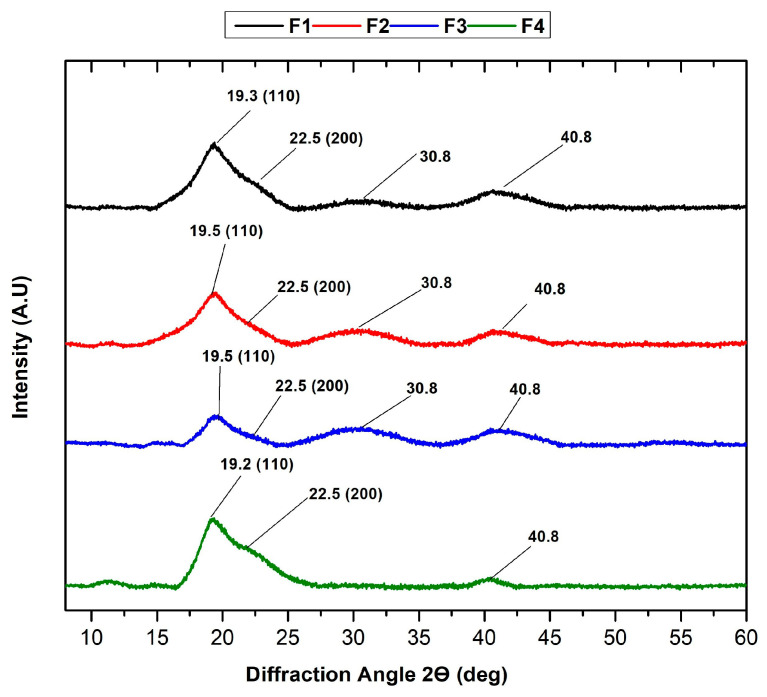
XRD analysis of PVA/CS/NPs-SiO_2_ scaffolds.

Figure 5 shows the crystallinity of the scaffolds, one of the main factors that influence the mechanical properties of the polymers. The amorphous nature of the PVA/CS polymer blend is evident. Amorphous diffraction peaks are reflected at 2θ = 19.3 and 22.5, attributed to the 110 and 200 planes, respectively [39], indicative of the PVA/CS blend [40]. FTIR analysis shows that OH groups are present in PVA and CS, thus forming solid inter- and intramolecular hydrogen bonds, reflected in the peak at 2θ = 40.8 [41]. A peak at 2θ = 30.8 is also observed in the first three formulations, possibly caused by the rearrangement of hydrogen bonds due to the fragmentation of the chitosan polymer chain [42]. With the addition of NPs-SiO_2_ and the decrease in the CS percentage in F2-F3, the diffraction intensity was narrower and weaker at peaks 19.5° and 22.5°. This is because the strong interactions between NPs-SiO_2_ and PVA alter the amorphous nature of the polymers, causing a decrease in intensity [43]. However, at F4, the intensity and peak width increase, possibly due to the high number of hydrogen bonds corresponding to the semi-crystalline structure of PVA, which may indicate higher amorphism in the scaffold structure [44].

As revealed through XRD analysis, the amorphous nature of nanomaterials offers several notable advantages. Amorphous materials often exhibit superior mechanical properties, such as increased toughness and flexibility, compared to their crystalline counterparts. This is because amorphous structures lack grain boundaries, which are common points of weakness in crystalline materials. Moreover, amorphous forms generally have higher solubility and bioavailability, which is crucial for biomedical applications where rapid dissolution and absorption can enhance therapeutic efficacy [45].

Furthermore, the uniform distribution of incorporated nanoparticles within an amorphous matrix leads to more consistent and predictable material properties. Additionally, amorphous materials typically show enhanced thermal stability, beneficial in high-temperature processing environments [46]. These characteristics make amorphous nanomaterials highly desirable for various applications, particularly in pharmaceuticals and tissue engineering.

#### 2.2.3. Thermal Analysis of PVA/CS/NPs-SiO_2_ Scaffolds

**Figure 6:**
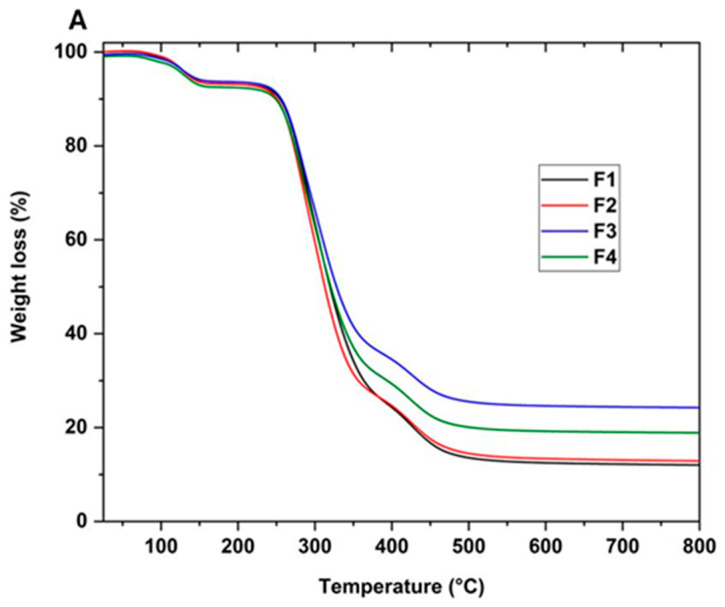

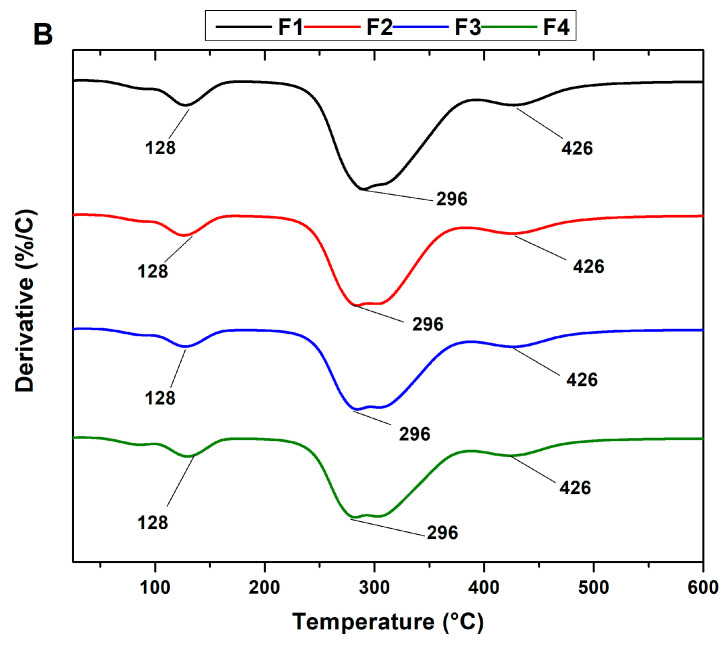
Thermogravimetric analysis of PVA/CS/NPs-SiO_2_ scaffolds. (**A**) Thermogram (TGA) and its derivatives (**B**) of scaffolds F1, F2, F3, and F4. PVA/CS/NPs-SiO_2_ scaffolds.

The thermal resistance of the scaffolds was studied using TGA analysis. Figure 6A shows the thermograms of the PVA/CS/NP-SiO_2_ scaffolds and Figure 6B derived from the thermograms. No differences in the three stages of degradation are observed in the thermograms. The first stage of degradation is attributed to the evaporation of the absorbed and physically bound water in the polymer matrix (between 100 and 150 °C). The second stage (between 200 and 400 °C) is associated with the disintegration of the PVA side chain and the decomposition of the CS polysaccharide unit. Finally, the third stage of degradation occurred between 400 and 480 °C, corresponding to the degradation of the CS glycosidic bonds and the degradation of the PVA polyene residues [47]. 

On the other hand, it was observed that the introduction of NPs-SiO_2_ did not alter the degradation stages in the formulations, keeping the maximum peaks similar. In F2, a slight reduction in mass loss temperature with a shift to the left between 200 and 300 °C is observed in the second stage. This shift suggests that nanoparticles are affecting the sample’s thermal decomposition. Nanoparticles are known to be resistant to high temperatures, which slows the weight loss in the sample [48]. As a result, in the third stage of degradation, between 300 and 400 °C, a slightly higher mass loss is observed in the F2 formulation compared to F1, which did not contain nanoparticles. This phenomenon suggests a higher mass retention in F2, attributed to the decrease in chitosan concentration and aggregation of nanoparticles. These changes promote multiple orientations between the polymer chains, contributing to slightly reducing the degradation temperature [48]. 

In the case of F3, a shift of the thermogram to the right is observed in the third stage, extending between 350 and 400 °C, leading to an increase in the mass loss temperature. A loss of 30% is recorded, indicating higher mass retention compared to formulations F1 and F2. This phenomenon is probably attributed to the increase in SiO_2_ nanoparticles and the decrease in chitosan in the mixture. This observation suggests a more significant interaction between the polymer matrix’s SiO_2_ nanoparticles and the -OH groups. This interaction strengthens the structure and promotes the formation of cross-links in the polymeric matrix, which improves the material’s thermal stability [49]. 

Finally, in F4, a 25% increase in material loss was observed in the third stage compared to F3. This phenomenon can be attributed to the excess of nanoparticles, which can lead to increased interference in the proper packing of the polymeric matrix. This interference hinders molecular packing and leads to a less uniform distribution, which may have implications on properties and exert an adverse effect on improving the thermal property of the scaffold [50]. 

**Figure 7:**
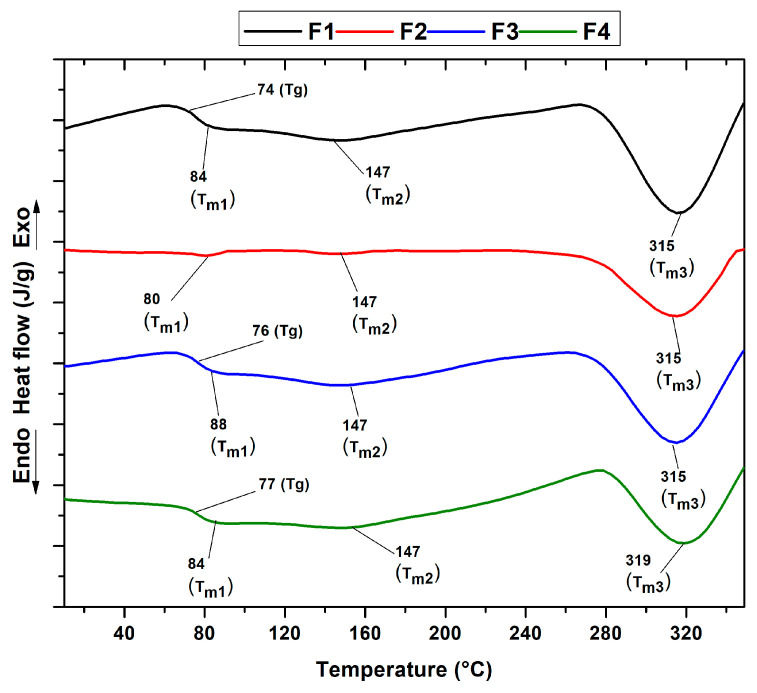
DSC curves of PVA/CS/NPs-SiO_2_ scaffolds.

As shown in Figure 7, differential scanning calorimetry (DSC) facilitated the analysis of the formulation’s thermal transitions. The different thermograms show the melting temperatures of chitosan (T_m1_) and PVA (T_m2_). Crystallization temperatures are not observed. Table 1 summarizes the thermal properties for each formulation, including the T_g_ and T_m3_, attributed to the predominance of PVA and the decomposition of the polymers.

Differential scanning calorimetry (DSC) analysis identified the thermal peaks in the four formulations, which showed minimal variation. A glass transition (T_g_) corresponding to the predominant PVA content was observed at 70 to 76 °C. This suggests a shift from a glassy to a more flexible state, as reported in previous studies [31,50]. The melting point T_m1_ characteristic of the semi-crystalline structure of chitosan ranged from 80 to 88 °C. Small fluctuations in the T_m1_ and T_g_ peaks were also detected, possibly associated with the moisture-retention capacity induced by the hydrophilic character of the silicon dioxide nanoparticles (NPs-SiO_2_), the plasticizing effect, and the remarkable chemical and thermal stability that these nanofillers provide when dispersed in the polymer matrix [51,52]. 

This finding is supported by the X-ray diffraction analysis of the PVA/CS mixture, which evidenced slight changes upon incorporation of the silicon dioxide nanoparticles. In addition, an endothermic peak (T_m2_) attributed to the melting process of the PVA/CS crystalline region was identified and observed at a temperature of 147 °C [30,31,33]. At higher temperatures, between 315 and 319 °C, a third pronounced peak corresponding to T_m3_ was observed, attributed to the decomposition of both the chitosan and the PVA side chains, an observation also confirmed in stage three of the thermogravimetric analysis (TGA) [37].

On the other hand, variations were detected in the intensity of the endothermic peak at F2, evidencing a decrease between 300 and 350 °C. This behavior could be attributed to the reduction in the percentage of chitosan and the introduction of silicon dioxide nanoparticles in the polymer matrix [53]. This intensity increased again in F3 and decreased in F4. This could be due to the decrease in the CS concentration and the presence of NPs-SiO_2_, which indicates high mobility between the polymeric chains and the generation of nucleation sites that increase the melting points as the scaffold degrades. X-ray diffraction analysis of the PVA/CS mixture, which changes slightly when silicon dioxide nanoparticles are incorporated, corroborates this result.

#### 2.2.4. Scanning Electron Microscopy (SEM) Of PVA/CS/NPs-SiO_2_ Scaffolds

**Figure 8:**
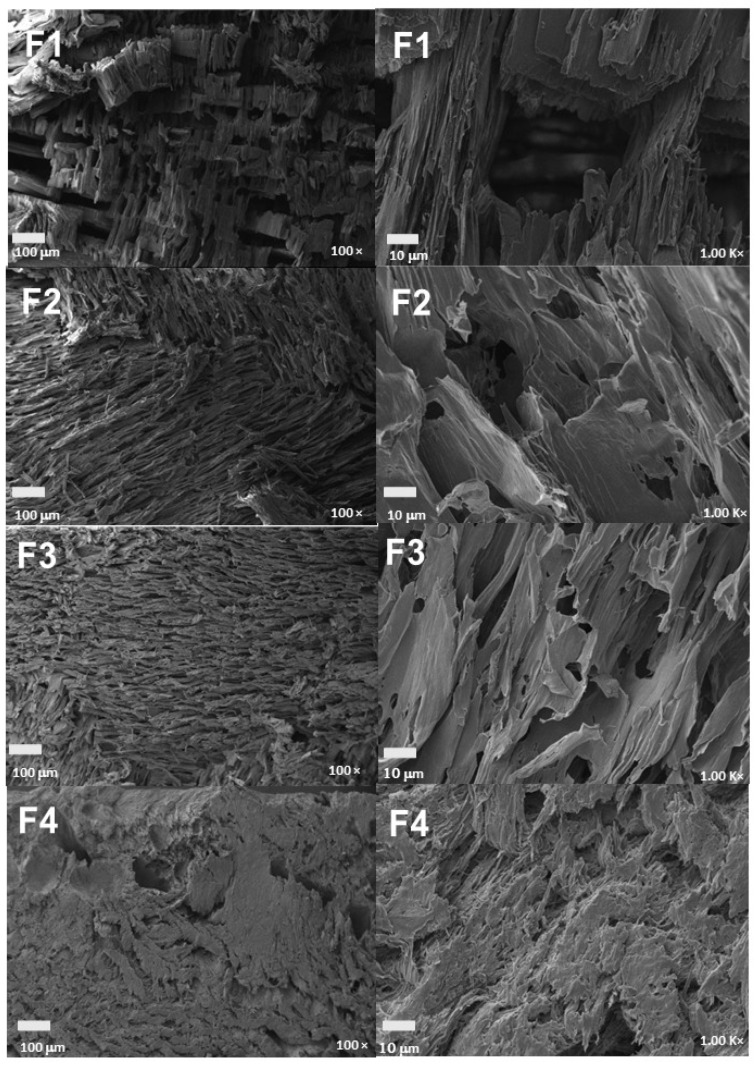
Morphology of PVA/CS/NPs-SiO_2_ scaffolds. F1, 70%PVA/30%CS at 100 y 1000×; F2, 70%PVA/29.5%CS/0.5%NPs-SiO_2_ at 100 and 1000×; F3, 70%PVA/29%CS/1%NPs-SiO_2_ at 100 and 1000×; F4, 70%PVA/28%CS/2%NPs-SiO_2_ at 100 and 1000.

The microstructure of the scaffolds is shown in Figure 8. A varied pore size ranging from 30–60 to 100–150 μm was evidenced due to the freeze-drying method, which generates a structure with ice crystals formed by the freezing of the water and acetic acid used that disappear later in the drying phase, leaving pores in the scaffold structure [54]. All four formulations showed porosity in their structure, which varied with the incorporation of nanoparticles. In particular, formulation F1 exhibited an inhomogeneous porous structure with multiple surface irregularities attributed to intermolecular interactions between the polymeric chains of CS and PVA [55]. However, the reduction in the CS percentage and the addition of nanoparticles in the mixture, as in formulation F2, showed significant improvements in the scaffold surface, with less presence of irregularities caused by the chelation reactions with the amino and hydroxyl groups of the chains of the PVA/CS mixture and the NPs-SiO_2_ [56]. 

The stability of silicon nanoparticles (NPs-SiO_2_) within the PVA/CS matrix, despite the relatively large pore size, is initially achieved by the strong hydrogen bonding interactions between the hydroxyl groups of PVA and the primary amino groups of CS with the surface of the NPs-SiO_2_, ensuring the anchoring of the nanoparticles firmly within the polymer matrix [57]. These molecular interactions are crucial to avoid the loss of the nanoparticles, ensuring their retention within the pores generated during the freeze-drying process. Subsequently, a more uniform morphology, with regular and homogeneous pores, was observed in formulations with higher percentages of NPs-SiO_2_, which act as reinforcements in the polymer matrix, increasing its strength and stiffness [58]. This incorporation of NPs-SiO_2_ not only contributes to the mechanical and thermal stability of the scaffolds but also improves the distribution and size of the pores, as evidenced in formulation F3, which increases the number, size, and distribution of the pores [59]. 

Finally, formulation F4 showed a more homogeneous and porous morphology, attributable to the strong interaction of the nanoparticles with the CS/PVA polymeric chains. In this formulation, the higher integration of NPs-SiO_2_ facilitates a more homogeneous pore distribution and a more robust structure, generating scaffolds with higher porosity and pores with more regular shapes and smaller diameters [60]. This indicates that, although the NPs-SiO_2_ are much smaller compared to the scaffold pores, their stability within the matrix is effectively maintained by molecular interactions and their role as structural reinforcements [61].

#### 2.2.5. Analysis of the Mechanical Properties Of PVA/CS/NPs-SiO_2_ Scaffolds

The mechanical versatility of polymeric biomaterials makes them particularly attractive in tissue engineering. Polymers can be engineered to have specific mechanical properties that resemble those of the natural tissue that is being regenerated. In addition, they can be chemically modified to improve their biocompatibility, degradability, and responsiveness to biochemical signals from the cellular environment [62]. The results of the mechanical tests on the four formulations of the PVA/CS/NPs-SiO_2_ scaffolds are detailed in Table 2.

The XRD analysis of PVA/CS/NPs-SiO^2^ scaffolds (Figure 5) shows the crystallinity of the scaffolds, which is one of the main factors influencing the mechanical properties of the polymers. The amorphous nature of the PVA/CS polymer blend is evident. This amorphous structure is advantageous as it generally results in superior mechanical properties such as increased toughness and flexibility. Amorphous materials lack grain boundaries, which are common points of weakness in crystalline materials, thus providing better durability and resilience under mechanical stress [45].

F1 (70%PVA/30%CS) serves as the baseline for comparison. It demonstrates moderate compressive strength with an elastic modulus of 0.0364 MPa, a maximum compressive stress of 0.575 MPa, and a high maximum strain capacity of 211.8%. These characteristics indicate that F1 has a homogeneous porous structure that absorbs compressive forces and allows significant deformation under load. This flexibility and moderate strength make F1 a viable scaffold for applications where substantial deformation is acceptable [62]. F2 (70%PVA/29.5%CS/0.5%NPs-SiO_2_) and F3 (70%PVA/29%CS/1%NPs-SiO_2_) significantly enhance mechanical strength due to NPs-SiO_2_.

For F2, the elastic modulus increases to 0.0455 MPa, and the maximum compressive stress rises to 1.028 MPa, although the maximum strain capacity decreases to 171.9%. For F3, the elastic modulus adjusts slightly to 0.0443 MPa, and the maximum compressive stress increases to 1.180 MPa, while the maximum strain capacity remains at 176.2%. These results suggest that the addition of nanoparticles in both formulations strengthens the scaffold and improves its stiffness, albeit at the cost of some flexibility [63]. The reinforcement effect of the NPs-SiO_2_ restricts the movement of the polymer chains, creating a denser and more rigid matrix that can withstand higher compressive stresses [63].

F4 (70%PVA/28%CS/2%NPs-SiO_2_) exhibits a different mechanical behavior. This formulation shows an increase in the maximum strain capacity to 243.4%, but the elastic modulus and maximum compressive stress decrease significantly to 0.00362 MPa and 0.3353 MPa, respectively. This change is likely due to the higher concentration of NPs-SiO_2_ and the reduction in CS in the mixture, leading to a less stiff but more flexible scaffold. SEM analysis supports this observation, revealing that F4 has a more compact and homogeneous structure with reduced internuclear spaces, allowing greater deformation under load [64]. 

The scanning electron microscopy (SEM) analysis confirms the structural observations for each formulation. F2 and F3 display similar morphologies with well-distributed NPs-SiO_2_, which contributes to their increased stiffness and strength. In contrast, F4 shows a more compact structure with a higher concentration of nanoparticles, reducing the internuclear spaces and enhancing the scaffold’s flexibility and deformation capacity under compressive stress [64]. These findings highlight the importance of tailoring the composition of PVA, CS, and NPs-SiO_2_ to achieve specific mechanical properties for different tissue-engineering applications. The inclusion of NPs-SiO_2_ not only reinforces the PVA/CS matrix, enhancing its strength and stiffness, but also improves its ability to maintain structural integrity under prolonged mechanical stress [60]. This is particularly crucial for applications in tissue regeneration, where scaffolds must withstand mechanical forces without collapsing, ensuring sustained function. Compared to commercial dressings such as PVA sponges or CS films, PVA/CS/NPs-SiO_2_ scaffolds demonstrate superior mechanical stability, making them highly suitable for tissue-regeneration applications that require robust mechanical properties [65,66].

A linear regression analysis was applied continuously to guarantee the reliability of the results in the three PVA/CS/NPs-SiO_2_ scaffold treatments. The analysis allowed evidencing low R^2^ values in Young’s Modulus (elastic), maximum compressive stress, and maximum deformation tests, where only 6.31%, 69.36%, and 19.85% of the data fit adequately to the linear model used. However, a one-way analysis of significant variance (ANOVA) with a 95% confidence level (5α = 0.05) was presented to test the validity of the linear model employed. Therefore, the acceptance of the null hypothesis is confirmed in the Young’s Modulus test, where the critical value of F = 0.18 is lower than the experimental value F = 0.907, indicating that there are no significant differences in the variation of the elastic capacity of the scaffolds between the formulations. In contrast, in the maximum compressive stress test, the critical value of F = 6.04 significantly exceeds the experimental value F = 0.019, suggesting significant differences between the treatments, and therefore the null hypothesis is rejected. On the other hand, in the maximum deformation test, the critical F = 0.66, unlike the experimental F = 0.599, suggests no significant differences between treatments, leading to the acceptance of the null hypothesis in this case. 

Additionally, the proposed linear model shows significant variation in the maximum compressive stress test for the F3 and F4 formulations, indicating a direct relationship between the increase in the percentage of NPs-SiO_2_ and the decrease in chitosan in the polymer matrix with the results obtained [66]. Consequently, it is concluded that the improved mechanical properties provided by NPs-SiO_2_ strengthen the scaffolds, granting them a greater capacity to resist structural collapse during dehydration in the freeze-drying process and, by extension, during their application in tissue regeneration [66]. These mechanical and structural properties not only ensure the durability of the scaffolds in the short term but also contribute significantly to their long-term stability and efficiency in biomedical applications, making PVA/CS/NPs-SiO_2_ scaffolds highly suitable to withstand the physiological environment and facilitate effective tissue regeneration.

### 2.3. Evaluation of the Antimicrobial Capacity of NPs-SiO_2_

The analysis of the minimum inhibitory concentration (MIC) made it possible to determine the inhibitory capacity of the NPs-SiO_2_ by selecting five Gram-positive and Gram-negative enterobacteria that were inoculated with nanoparticle solution at 37 °C for 24 h to determine the lowest concentration at which turbidity was not observed, thus confirming the inhibition of bacterial biological activity (Table 3).

The microorganisms *E. cloacae*, *E. coli*, *K. pneumoniae*, and *S. enterica* ATCC 53648 belong to the group of Gram-negative bacteria, which have a skinny peptidoglycan layer, and an outer layer consisting of lipopolysaccharides, lipoproteins, and lipids [67]. The minimum inhibitory concentration for *E. cloacae* and *E. coli* was 3.13 mg/mL because NPs-SiO_2_ can release silicon ions. These ions bind to the cell membrane through electrostatic interactions, attacking the peptidoglycan layer, altering membrane permeability [68], and leading to the destruction of the bacterial envelope. Additionally, the nanoparticles inhibit protein synthesis and reduce the activity of respiratory enzymes in the cytoplasmic membrane [69], blocking ATP production in the cell by interacting with amino groups (NH_2_) and carboxyl groups (COOH) of the membrane proteins, which induces cell apoptosis [70,71].

However, *K. pneumoniae* and *S. enterica* ATCC 53648 presented a high minimum inhibitory concentration of 25.0 mg/mL. This difference is attributed to the composition of their cell membrane, which contains lipopolysaccharides and phospholipids with a high number of hydroxyl and phosphate functional groups, providing an overall negative charge to the cell membrane [72,73]. On the contrary, the NPs-SiO_2_ synthesized by the sol-gel method attributed to them a slight negative charge generated by the presence of siloxane and silanol. Consequently, these negative charges produce high levels of repulsion and, in turn, decrease nanoparticle–bacteria interactions [74,75].

For *S. aureus* ATCC 55804, a Gram-positive bacterium with a thick peptidoglycan layer in its cell wall, no MIC data were obtained, probably due to the ability to resist treatment with the scaffolds. Multiple studies have documented the efficiency of NPs-SiO_2_ against enterobacteria, including *Escherichia coli* and *Staphylococcus aureus*, seeking to reduce the pathologies produced by these microorganisms in the skin and mucosal areas [76,77]. 

For this reason, research continues to demonstrate the antimicrobial properties and biocompatibility of these nanomaterials and their possible uses in different medical fields.

### 2.4. Antimicrobial Testing of PVA/CS/NPs-SiO_2_ Scaffolds

Four bacterial strains, *K. pneumoniae*, *E. cloacae*, *S. enterica* ATCC 53648 (Gram-negative), and *S. aureus* ATCC 55804 (Gram-positive), were used to test the compounds’ antimicrobial activity (Table 4).

The results presented concerning bacterial inhibition can be observed. For F1, an inhibition of 99.88% was given for *K. pneumoniae*, *E. cloacae*, and *S. aureus* ATCC 55804, where the inhibitory effect exerted by the PVA/CS mixture can be evidenced, attributed to the hydrophobic character and the chelating capacity of CS together with the interactions of the amino groups with the bacterial membrane [78]. The highly charged nature of the lipopolysaccharides confers an overall negative charge to the cell wall, which facilitates their interaction with the positively charged amino groups of the CS, causing alterations in permeability and a loss of components and nutrients, thus confirming the considerable antibacterial action against the microorganisms [79,80]

On the other hand, for F2 and F3, there was an increase in the inhibitory capacity of the *K. pneumoniae* strain, with 100% inhibition. Initially, this is due to the presence of protonated amino groups in the chitosan that confers a positive charge to the scaffold, helping the interaction with the active sites in the cell membrane containing sulfur and phosphorus, producing damage to the cell wall [81]. In addition, NPs-SiO_2_ in F2 and F3 are adsorbed by the phospholipid membrane, damaging fluid transport across the bilayer and resulting in efficient antibacterial inhibition.

Compared to commercial dressings and materials reported in the literature, PVA/CS/NPs-SiO_2_ scaffolds offer significant advantages due to their built-in antimicrobial capacity. Many commercial dressings do not possess inherent antimicrobial agents and rely on the addition of antibiotics or other antimicrobial materials [82]. On the other hand, PVA/CS/NPs-SiO_2_ scaffolds combine superior mechanical properties, biocompatibility, and effective antimicrobial capacity in a single structure. This positions them as an advanced option for wound healing, providing both structural support and antimicrobial protection without the need for additional treatments [83].

Although F4 showed a decrease in the inhibition ability for *S. aureus* ATCC 55804, with a value of 82%, this formulation still demonstrates considerable efficiency against Gram-positive bacteria. This may be attributed to the thick peptidoglycan layer of *S. aureus* ATCC 55804 and its intrinsic resistance, which hinders the interaction of the NPs-SiO_2_ with the cell wall [84]. PVA/CS/NPs-SiO_2_ scaffolds stand out not only for their ability to effectively inhibit a wide range of bacteria but also for integrating antimicrobial properties into a mechanically robust and biocompatible structure. This combination of features makes them highly competitive and potentially superior to other commercial wound dressings and materials reported in the literature [77,82]. 

This combination of chitosan as a biodegradable biopolymer with antibacterial properties and various applications in pharmaceuticals, medicine, wastewater treatment, biotechnology, cosmetics, the textile industry, and agriculture supports the findings by demonstrating that encapsulation with chitosan enhances the stability and bioavailability of goji berry and garlic compounds [80]. Chitosan provides antimicrobial activity, efficacy, and low cytotoxicity along with NPs-SiO_2_, making it an ideal option for applications requiring both biocompatibility and antimicrobial protection.

Silicon dioxide nanoparticles (NPs-SiO_2_) are highly effective in antimicrobial applications due to their ability to disrupt bacterial cell walls and inhibit growth [85]. Despite this strong antimicrobial activity, NPs-SiO_2_ exhibit low cytotoxicity toward human cells, making them suitable for biomedical applications such as tissue engineering and wound healing [83]. This selective non-cytotoxic action is attributed to the fundamental differences between bacterial and human cell structures [86]. Bacteria have a peptidoglycan layer in their cell walls, which is a primary target of NPs-SiO_2_, whereas human cells lack this layer, possessing more complex cell membranes that offer protection against nanoparticle interaction [87]. Studies support the biocompatibility of NPs-SiO_2_, showing minimal cytotoxic effects on human cells while maintaining strong antimicrobial properties [88]. For instance, Handral et al. reported minimal cytotoxicity of NPs-SiO_2_ on human fibroblast cells, combined with effective bacterial inhibition [89]. Similarly, Colilla et al. observed that NPs-SiO_2_ maintained over 80% cell viability in human epithelial cells at higher concentrations alongside robust bacterial growth inhibition [90]. Fonseca et al. further highlighted that NPs-SiO_2_ cause lysis in bacterial cells without significant effects on human cells, underscoring their selective action based on structural differences [91]. This combination of high antimicrobial efficacy and low cytotoxicity makes NPs-SiO_2_ an ideal choice for applications requiring both biocompatibility and antimicrobial protection [92,93,94].

### 2.5. In Vivo Biocompatibility Testing of PVA/CS/NPs-SiO_2_ Scaffolds

**Figure 9:**
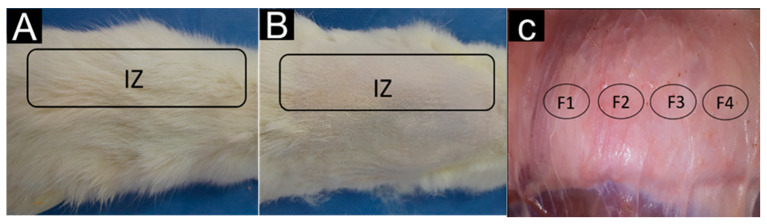
Implanted areas in the skin of Wistar rats. (**A**): Dorsal area with hair regrowth. (**B**): Dorsal area with trichotomy. (**C**): Internal surface of the dorsal area. IZ: implantation zone. F1: implantation zone formulation F1. F2: implantation zone formulation F2. F3: implantation zone formulation F3. F4: implantation zone formulation F4.

Macroscopic inspection of the samples indicated that at 60 days, there was complete hair recovery on the dorsal surface of the biomodels, as shown in Figure 9A. After performing a trichotomy of the skin, the area was observed to be completely healed (Figure 9B). In addition, when checking the inner part of the skin, it is not possible to see the implantation areas, but they were palpable as minor incursions inside the tissue. These initial observations suggest that the materials used are biocompatible.

Rats are widely used in experimental healing designs because this process is similar in all mammals. They offer the advantage of cost, size, and ease of handling [95]. In addition, healing in rats occurs mainly through a contraction, which makes them faster [96]. The areas where the incisions were made had usually healed. In the internal part of the skin, the areas where the material was implanted were practically indistinguishable from areas without intervention. This gives the first idea that the materials used were biocompatible.

**Figure 10:**
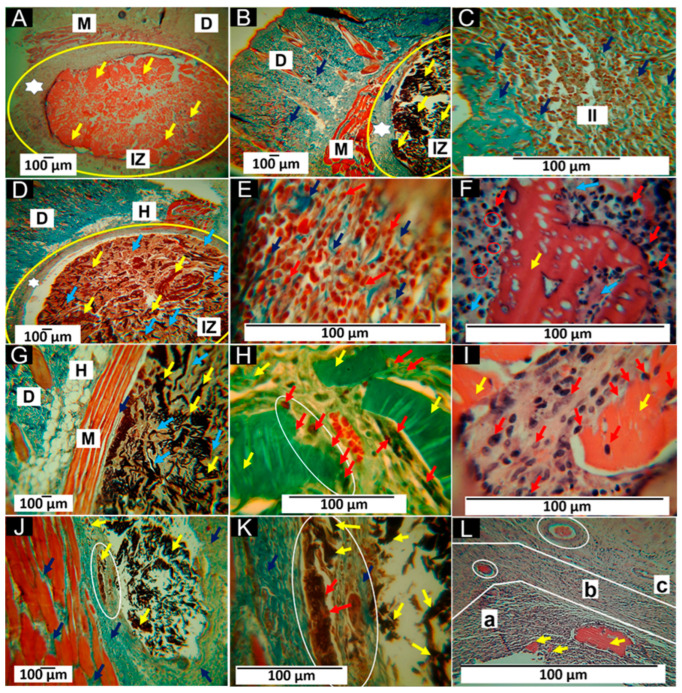
Scaffolds implanted at 60 days. (**A**): 4× image, HE technique. (**B**): 4× image, MT technique. (**C**): 40× image, MT technique. (**D**): 4× image, MT technique. (**E**): 100× image, MT technique. (**F**): 100× image, HE technique. (**G**): 4× image, MT technique. (**H**): 40× image, GT technique. (**I**): Image at 100×, HE technique. (**J**): 4× image, MT technique. (**K**): Image at 40×, MT technique. (**L**): Image at 100×, HE technique. M: muscle. D: dermis. H: hypodermis. IZ: implantation zone. Yellow oval: implantation zone. Red oval: multinucleated cells in a disordered arrangement, which appear partially engulfing and adapt their morphology to foreign body particles, as seen in (**F**). White star: fibrous cap; yellow arrows: scaffold fragments. Dark blue arrows: collagen type I. II: inflammatory infiltrate. Red arrows: inflammatory cells. Light blue arrows: collagenous matrix deposits. White ovals: areas of histological interest. a: Area “a”. b: Area “b”. c: Area “c”. (**A**–**C**) correspond to formulation F1. (**D**–**F**) correspond to formulation F2. (**G**–**I**) correspond to formulation F3, and (**J**–**L**) correspond to formulation F4.

Figure 10 shows the histological study of the four formulations. Figure 10A corresponds to a completely fragmented and encapsulated structure. The implantation zone (IZ) is highlighted with a yellow oval. A wide capsule surrounds the IZ in that zone, and material is present as multiple fragments marked with yellow arrows. In Figure 10B, type I collagen fibers are observed with the MT-staining technique, indicated by dark blue arrows in the fibrous capsule (white star). Figure 10C corresponds to the interface between the fibrous capsule and the fragments of the material. The capsule area is blue due to the presence of type I collagen, and in the area of the material fragments, there is a moderate inflammatory infiltrate; the presence of type I collagen fibers in the middle of this infiltrate is also highlighted.

Formulation F2 had the percentage of CS slightly decreased, and 0.5% of NPs-SiO_2_ was incorporated. This change seems to have influenced the behavior of the material. Figure 10D shows a decrease in the thickness of the fibrous capsule that delimits the implantation zone. The fragmented structure with a deposit of connective tissue matrix in the middle of the fragments is also observed (light blue arrows). The interface zone between the fibrous capsule and the fragment zone is similar to that reported for formulation F1. Figure 10E shows an inflammatory infiltrate (red arrows) with type I collagen fibers (dark blue arrows). In Figure 10F, a large fragment of the partially degraded material is observed being attacked by phagocytic cells (red arrows). Additionally, the presence of multinucleated cells is highlighted with red circles.

About formulation F3, it should be noted that the fibrous capsule has been replaced by a few collagen fibers, which can be observed between the muscle and the material in Figure 10G, indicated with a dark blue arrow. An increase in the connective tissue matrix where the fragments of the material are immersed is also observed (light blue arrows). The Gomori staining technique (GT) identified type III collagen in the connective tissue matrix, as seen in Figure 10H. In this image, blood vessels and a group of cells phagocytizing a fragment of the scaffold (white oval) can also be observed. In Figure 10I, one can see how the pieces of material are attacked by the phagocytic cells (red arrows). In addition, reabsorbing cells can be found at the periphery of the fragment and in some surface spaces where pores were formed, possibly by hydrolytic degradation.

The results for formulation F4 indicate a more advanced degradation and resorption process than the other formulations. The fibrous capsule is no longer present, and a collagen matrix occupies the spaces where the material was. Figure 10J, obtained by Masson’s staining technique (MT), shows the presence of blue fibers identified as type I collagen (dark blue arrows) and other unstained fibers possibly corresponding to type III collagen. Some tiny fragments of the matrix are also present in this collagen matrix.

Figure 10K is taken at 40× magnification with the MT technique and shows some small fragments in the collagen matrix as they are resorbed. Figure 10L approximates how the material is resorbed and simultaneously replaced by a connective tissue matrix. The image, which corresponds to a 100× magnification of an area where most of the material has been resorbed, allows us to identify three regions with different histologic appearances: the area identified as “a” corresponds to the resorption of larger fragments (yellow arrows), with abundant presence of inflammatory cells, where the white spaces between the cells correspond to deposits of collagenous matrix. The area identified as “b” corresponds to a site with a much more advanced resorption process of the material. On the other hand, in the white circle, a small fragment without resorption is highlighted. In addition, there is a lower density of cells and more excellent occupation of the spaces between cells in the connective tissue matrix stained with a light purple color. In zone “c”, there is also a fragment of non-resorbed material (white circle). Still, the cell density is markedly lower, and a more significant amount of connective tissue matrix is evident.

The histological studies show differences in the porous scaffolds’ in vivo behavior. The four implanted formulations comprise PVA and CS, compatible materials with different biological properties. CS has been used in multiple investigations due to its resorbability and ability to promote tissue healing by promoting cell differentiation and proliferation [97]. However, its poor mechanical properties make it necessary to functionalize it with other compounds [98].

The other component (PVA) has been used in various applications, such as scaffolds and films, to improve the mechanical properties of CS [99]; in addition, it presents properties of biological interest such as biocompatibility, hydrophobicity, and water solubility [100]. The degradation of PVA is mainly hydrolytic, while CS’s is biological [101]. This explains why, at 60 days, the material is so fragmented and surrounded by a fibrous capsule, as observed in Figure 10A. The high presence of phagocytic cells summoned to eliminate all the material remains (Figure 10C). The histological images in Figure 10A–C, corresponding to the scaffolds with a PVA70%/CS30% composition, correspond to a healing process with a foreign body reaction (FBR), fibrous capsule, and an inflammatory infiltrate. Despite its biocompatibility, it is accepted that CS can induce a mild and moderate transient FBR [102].

The foreign body reaction in itself is not bad. It occurs because of a failed transition between the inflammatory and proliferative phases of healing. It is due to a material that has not been completely reabsorbed [100]. It is considered a normal body response to an implanted material [103]. FBR is the organism’s mechanism to control damage and prevent potentially toxic degradation products from damaging neighboring tissues. This leads to the granulation tissue that initially forms being transformed into less cellular tissue with a higher collagen content [104], as seen in Figure 10A.

In formulation F2, the amount of PVA remained constant, but CS was decreased by 0.5%, and 0.5% of NPs-SiO_2_ was incorporated. The presence of PVA ensures the fragmentation of the material by hydrolytic action, as seen in Figure 10D. As CS decreases, the degradation and resorption of the scaffold is also faster, where NPs-SiO_2_ seems to have influenced the deposition of more extracellular matrix (Figure 10E), which can be explained by the ability of these nanoparticles to act with other cells and the environment. However, NPs-SiO_2_ (like other nanoparticles) are not degradable and must be eliminated via cellular phagocytosis [105]. This would explain the presence of inflammatory cells attacking the material (Figure 10E); additionally, multinucleated cells in a disordered arrangement are highlighted with red circles, which appear partially engulfing and adapt their morphology to foreign body particles, as seen in Figure 10F.

In formulation F3, the PVA remains constant, which guarantees the scaffold’s fragmentation (Figure 10G), but the percentage of CS decreased to 69%, and the rate of nano-particles increased to 1%. These two changes caused higher material resorption and the need to phagocytize the scaffold fragments, as Figure 10H,I observed. Blood vessels are also highlighted, although these are always present in the healing process.

The results observed after the implantation of formulation F4 are different from those of the other three formulations. The fibrous capsule has been replaced by connective tissue that grows simultaneously with the degradation and resorption of the material, as shown in Figure 10J,K. Figure 10L lets us understand how a connective tissue matrix replaces the material with type I and possibly type III collagen fibers, restoring the affected tissue.

The results of the four formulations showed that the scaffolds were compatible and resorbable. The degradation and absorption process combines the hydrolytic degradation pathway by the presence of PVA and the cellular enzymatic pathway by CS and the presence of the NPs-SiO_2_, resulting in a progressive replacement of the implanted material by maturing connective tissue. SiO_2_ is considered a biocompatible material, and the NPs-SiO_2_ have had wide biomedical applications in different fields due to their biological qualities [106,107].

In the case of this work, the NPs were present in small amounts and functionalized with the other components. The results show that the percentages used did not present a cytotoxic effect, and an increase in biocompatibility was observed, which was reflected in the disappearance of the fibrous capsule and the replacement of the implanted material with a more mature connective tissue matrix, as observed in the results of formulation F4. Compared to other wound dressings, PVA/CS/NPs-SiO_2_ scaffolds not only offer superior biocompatibility and improved tissue integration but also provide controlled resorption and effective promotion of tissue regeneration, aspects that are critical for their application in wound healing.

## 3. Materials and Methods

### 3.1. Materials

**Figure 11:**
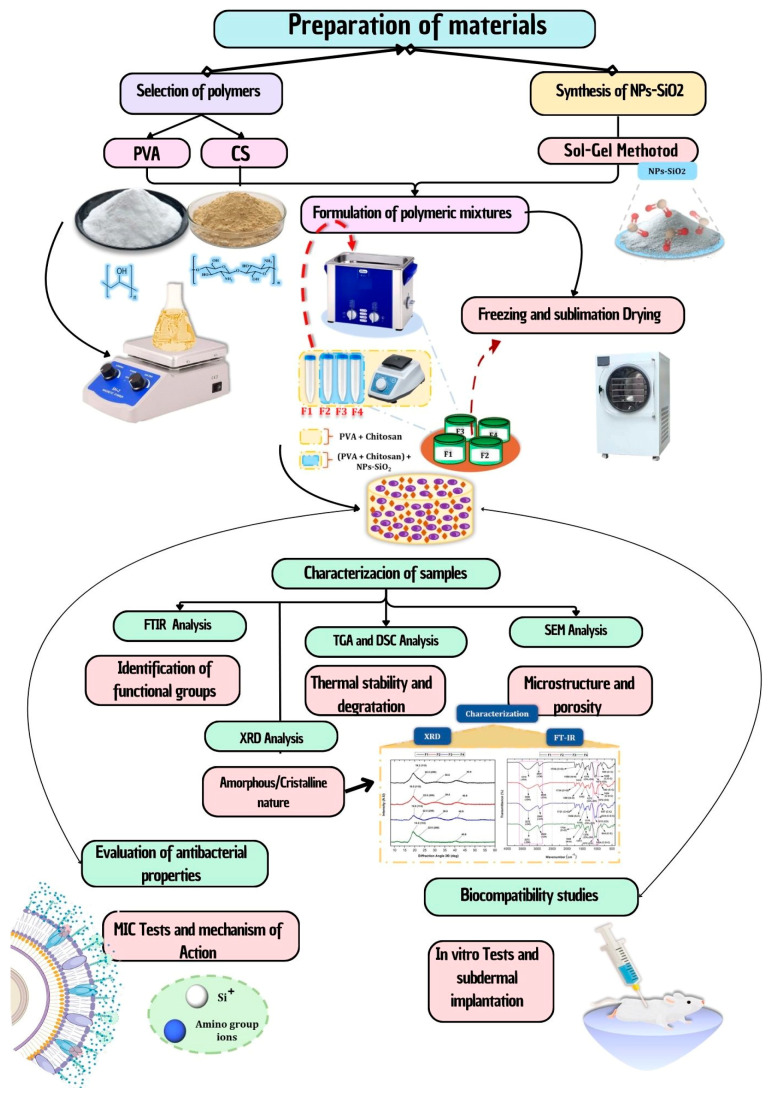
Flowchart of the methodology for preparing and characterizing PVA/CS/NPs-SiO_2_ scaffolds. Includes selection of polymers (PVA and CS), synthesis of NPs-SiO_2_, formulation of polymeric mixtures, freeze-drying method, and characterization of samples (FTIR, TGA, DSC, SEM, and XRD). The image also details the evaluation of antibacterial properties and biocompatibility studies.

The chemical reagents used in the investigation were of analytical grade and without purification unless otherwise indicated (Figure 11). All reagents used in this study were obtained from Sigma, Aldrich (St. Louis, MO, USA). The chitosan (CS) used had a low molecular weight (1 × 10^6^ Da), a degree of deacetylation of >80%, and a viscosity of 20–300 cP (in 2% acetic acid), depending on the supplier, while polyvinyl alcohol showed 90% hydrolysis and contained 95.000 g/mol. Hexadecyltrimethylammonium was obtained from CTAB, Panreac (Barcelona, Spain), and tetraethyl orthosilicate was purchased from TEOS, Sigma Aldrich (Darmstadt, Germany). Finally, the nanoparticles were calcined in a Nabertherm LHT 02/18 furnace (Lilienthal, Bremen, Germany).

### 3.2. Synthesis of Silicon Dioxide Nanoparticles (NPs-SiO_2_)

The synthesis of the nanoparticles was carried out using the previously reported methodology [108]. It was carried out in an aqueous solution of 400 mL of hexadecyltrimethylammonium (CTAB) and 16 mL of NH_4_OH to obtain basic pH. The volume was then made up to 500 mL with H_2_O, 17.14 mL of tetraethylorthosilicate (TEOS) was added by drip with 700 rpm agitation, and the temperature was raised to 95 °C for one hour. Next, the NPs-SiO_2_ were centrifuged at 5000 rpm for 15 min and washed with ethanol and distilled water in triplicate to remove any remaining hexadecyltrimethylammonium. Finally, the nanoparticles were calcined in a Nabertherm LHT 02/18 furnace (Lilienthal, Bremen, Germany) at 250 °C.

### 3.3. Characterization of Silicon Dioxide Nanoparticles (NPs-SiO_2_) and Scaffolding PVA/CS/NPs-SiO_2_

Several advanced techniques were employed to characterize silicon dioxide nanoparticles (NPs-SiO_2_) and PVA/CS/NPs-SiO_2_ scaffolds.

#### 3.3.1. Fourier Transform Infrared Spectroscopy (FTIR)

Fourier Transform Infrared Spectroscopy (FTIR) was used to identify the functional groups and chemical bonds in both NPs-SiO_2_ and the PVA/CS/NPs-SiO_2_ scaffolds. The NPs-SiO_2_ were analyzed with a Shimadzu FTIR-8400 spectrophotometer (Shimadzu, Kyoto, Japan) featuring a diamond tip accessory, and the scaffolds were analyzed with a Shimadzu IR Affinity-1 spectrophotometer (Shimadzu, Kyoto, Japan), both covering a wavelength range of 500–4000 cm⁻¹ [109]. This technique highlighted the presence of Si-O-Si bonds in the nanoparticles and the O-H, C-H, and Si-O bonds in the scaffolds, confirming the successful integration of NPs-SiO_2_ into the polymer matrix

#### 3.3.2. X-ray Diffraction (XRD)

X-ray Diffraction (XRD) was employed to determine the crystalline structure and phase composition. Both NPs-SiO_2_ and scaffolds were examined using a PANalytical X’Pert PRO diffractometer (Malvern Panalytical, Royston, UK) with Cu Kα radiation (1.540598 Å for Kα1 and 1.544426 Å for Kα2) operating at 45 kV. The analysis spanned a 2θ range from 5° to 80°, providing clear insights into the crystalline phases of NPs-SiO_2_ and the diffraction patterns of the composite scaffolds.

#### 3.3.3. Electron Microscopy TEM and SEM

For morphological analysis, Transmission Electron Microscopy (TEM) was used to examine the NPs-SiO_2_. This was conducted with a JEOL ARM 200 F microscope (JEOL Ltd., Tokyo, Japan) operated at 20 kV. The average diameter of 100 nanoparticles was measured using Image J software, revealing a narrow size distribution. The surface morphology of the PVA/CS/NPs-SiO_2_ scaffolds was studied using Scanning Electron Microscopy (SEM). The analysis was performed with a Hitachi TM 3000 microscope (Hitachi High-Technologies Corporation, Tokyo, Japan) in secondary electron mode at 20 kV. The scaffolds were gold-coated to enhance electron conductivity, and the SEM images demonstrated a porous structure with homogeneously distributed NPs-SiO_2_.

#### 3.3.4. Analysis of the Mechanical Properties of PVA/CS/NPs-SiO_2_ Scaffolds

Compression tests were performed to analyze the mechanical properties of the scaffolds using a SHIMADZU EZ-LZ universal testing machine (Shimadzu, Tokyo, Japan). This machine was operated with a 500 N load cell. Three specimens were taken for each formulation and treated in triplicate at 10 mm/min speed, with a jaw spacing of 10 mm and a scaffold width of 20 mm.

#### 3.3.5. Thermal Analysis Of PVA/CS/NPs-SiO_2_ Scaffolds

For thermogravimetric analysis (TGA), a NETZSCH TG 209 F1 Libra (Mettler Toledo, Schwerzenbach, Switzerland) was used, with a temperature of 10 °C/min between 25 and 800 °C under nitrogen atmosphere (10 mL/min). Thermal transitions such as glass transition temperature (T_g_), melting temperature (T_m_), and crystallization temperature (T_c_) were determined by differential scanning calorimetry (DSC) using a DSC1/500 apparatus (Mettler Toledo, Schwerzenbach, Switzerland). The scaffolds were heated between −25 °C and 250 °C, with a heating rate of 10 °C/min and a nitrogen flow rate of 60 mL/min. All DSC data were reported from the second heating sweep, seeking to eliminate the thermal history of the polymers. The data obtained from the TGA and DSC were analyzed using TA Instruments Universal Analysis Software 2000 version 4.5 [110].

### 3.4. Synthesis of PVA/CS/NPs-SiO_2_ Scaffolds

To prepare the PVA/CS/NPs-SiO_2_ scaffolds, 6 g of CS was initially dissolved in 300 mL of 2% acetic acid and heated to 40 °C. Next, 16 g of PVA was weighed and dissolved in 200 mL of distilled water at 80 °C [111]. Subsequently, 200 g of NPs-SiO_2_ was weighed and dispersed in 20 mL of distilled water with an ultrasonic bath (Branson, Madrid, Spain) for one and a half hours. Each component was mixed in the corresponding amount until a final concentration of 4% (*w*/*v*) was obtained (Table 5). The resulting mixture was placed in an ultrasonic bath for 10 min to eliminate air bubbles in the solution. Subsequently, they were added to previously sterilized cylindrical glass containers to be taken to the freeze-drying process in an LC-FD-06H freeze-dryer (Müller Scientific, Zhengzhou, China), where a vacuum was applied with a 2XZ-4 rotary vane vacuum pump (East Vacuum, Beijing, China) until reaching 5.5 Pa for 72 h, obtaining the PVA/CS/NPs-SiO_2_ scaffolds [109,110].

### 3.5. Evaluation of the Antimicrobial Capacity of NPs-SiO_2_

A 50 mg/mL stock solution was prepared with the SiO_2_ nanoparticles by taking 250 mg of NPs-SiO_2_ and diluting it in 5 mL of distilled water. The NPs-SiO_2_ were dispersed by applying vortex agitation and 2 min of ultrasound. Six successive dilutions (3.0 mL of the above stock and 3.0 mL of distilled water) were prepared (50.0, 25.0, 12.5, 6.25, 3.125, 1.56, and 0.78 mg/mL). Five bacteria were selected (*Salmonella enterica* ATCC 53648, *Staphylococcus aureus* ATCC 55804, *Klebsiella pneumoniae*, *Enterobacter cloacae* sub cloacae, and *Escherichia coli*) for the antimicrobial tests previously identified molecularly, selected from the culture collection of the Universidad de San Buenaventura Cali. Enterobacteria were grown in BHI broth for 24 h and washed 3 times with sterile 0.85% saline by vortex shaking for 1 min and centrifugation at 6000 rpm for 5 min. From the final solution, dilutions were made until the concentration was adjusted to 1 × 10^5^ CFU/mL using the optical density value (OD 600) with the McFarland scale, using TSB broth (tryptase soy) [112]. In a 96-well polystyrene microplate (BRANDplates^®^, Wurtemberg, Germany), 100 µL of the enterobacteria and 100 µL of the nanoparticle dilution were inoculated. The microplates were incubated at 37 °C for 24 h. The minimum inhibitory concentration (MIC) value was determined as the lowest concentration at which no turbidity or bacterial growth was observed. To confirm biological activity, 20 µL of 0.2% triphenyl tetrazolium chloride (TTC) solution was added and incubated at 37 °C for two hours; red indicates biological activity [113].

### 3.6. Antimicrobial Testing of PVA/CS/NPs-SiO_2_ Scaffolds

Fifty milligrams of the scaffold was deposited in a test tube with 5.0 mL of sterile distilled water. It was left to stand for 2 h and vortexed for 1 min. Then, 100 µL of the previously adjusted strains was added at a concentration of 1 × 10^6^ CFU/mL and vortexed for 1 min. 

The tubes were incubated at 37 °C for 24 h. The control was performed for each of the strains using 0.85% saline and without the addition of the scaffolds. At the end of the incubation time, viable cells were counted using the plate count technique [114]. Briefly, at the end of the incubation time, tubes of each treatment were vortexed for 1 min, successive dilutions were made in 0.85% saline, and 100 µL was seeded on plates with nutrient agar. The plates were incubated at 37 °C for 48 h. Determinations were performed in triplicate. The reduction in viable cells due to the scaffold compared to the no-scaffold control was reported as percentage inhibition of the material.

### 3.7. Preliminary In Vivo Biocompatibility Analysis of PVA/CS/NPs-SiO_2_ Scaffolds

Subdermal Surgical Implantation of Biomodels. For the in vivo biocompatibility test of the 4 formulations, subdermal implantations were performed in three male Wistar rats, 5 months old and weighing an average of 390 g.

The biomodels were randomly selected from the murine population of the LABBIO laboratory of the Universidad del Valle and remained in this laboratory for the entire research duration. The surgical design used the subdermal implantation model that allows for the simultaneous testing of several formulations [115]. During surgery, sedation was performed with Ketamine 70%/Xylazine 30% in weight-adjusted doses (Ketamine 50, Holliday Scott Laboratory, Béccar, Buenos Aires, Argentina), Xylazine (ERMA Laboratories, Celta, Colombia), and, subsequently, trichotomy of the dorsal surface was performed, disinfected with iodopovidone (isodine solution, Sanfer Laboratories, Bogota, Colombia). Subsequently, local anesthesia was performed using 2% lidocaine with epinephrine (NewStetic Laboratory, Guarne, Antioquia, Colombia). For the implantation, each model underwent four incisions of 5 mm in length and total thickness on the dorsal surface of the skin. Four pocket-type surgical preparations were created using a blunt instrument 2 cm deep and 5 mm in diameter. Subsequently, a fragment of each type of scaffold was deposited in each of the preparations; each fragment had an average weight of 0.0304 g, and the preparation was closed with absorbable suture caliber 4 zeros (Vicryl Ethicon, Johnson & Johnson Laboratories, Cali, Colombia).

### 3.8. Histological Tests

Once the 60-day implantation period was completed, the biomodels were euthanized via intraperitoneal application of a solution of Ketamine 90%/Xylazine 10% with excess dose. Initially, a visual inspection of the intervened areas was performed. The samples were recovered and fixed with buffered formalin for 48 h. Subsequently, they were washed for 15 min with a phosphate-buffered solution, and the samples were prepared for histological purposes. Sample processing consisted of dehydration, diaphanization, and paraffinization using Leica TP 120 equipment (Leica Microsystems, Mannheim, Germany). For paraffinization, the samples were embedded in kerosene blocks with the Thermo Scientific™ Histoplast Paraffin™ kit (Fisher Scientific, Waltham, MA, USA), and 6-micron-thick kerosene sections were obtained using the Leica RM 2135 rotation microtome. Staining was performed using Hematoxylin–Eosin (HE), Gomori trichrome (GT), and Masson (MT) techniques to visualize the histological structures in the sections. The Leica DM750 optical microscope was used for image analysis, with the Leica DFC295 built-in camera and the image management program Leica Application Suite version 4.12.0 software (Leica Microsystem, Mannheim, Germany). 

The ethical component of this research was reviewed, approved, and supervised by the Committee for the Supervision of Biomedical Experimental Animals of the Universidad del Valle in Cali, Colombia, using resolution CEAS 006-022, 5 September 2022. The recommendations of the Animal Research: Reporting of In vivo Experiments—The ARRIVE guidelines [116] were followed for reporting the procedures performed. In none of the research phases were there any complications related to the biomodels, nor deaths of the biomodels. In the design of the experimental model and the development of the research, the principles of the three “Rs” [116] were applied, using the minimum number of animals recommended by the ISO 10993 standard [117] to adhere to the principle of “Reduction”, and standardized operating protocols were followed (principle of “Refinement”).

### 3.9. Statistical Analysis

For all the mechanical and antimicrobial test results, an analysis of variance (ANOVA) was applied to compare the mean of the three trials by pooling information using Fisher’s LSD method to determine significant differences. A linear regression analysis was applied to ensure the reliability of the results obtained in the three PVA/CS/NPs-SiO_2_ scaffold treatments. Minitab 19 software was used to analyze variance with a significance level of 0.05.

## 4. Conclusions

This study used the freeze-drying method to synthesize four membrane formulations of PVA and chitosan with SiO_2_ nanoparticles. NPs-SiO_2_ were incorporated into the polymeric matrices used. These membranes were characterized by infrared spectroscopy (FTIR), X-ray diffraction (XRD), thermogravimetric analysis (TGA), and differential scanning calorimetry (DSC). The FTIR analysis allowed evidencing the OH, C-O, C-H, and C=O vibrational peaks to provide the distinctive bands of PVA and CS polymers. A broad band is observed between 3272 and 3316 cm^−1^, indicating the presence of hydrogen bonds between the chitosan and polyvinyl alcohol, causing stretching and OH/NH_2_ overlapping, which presented changes as the percentage of chitosan decreased and the nanoparticles were incorporated. This may be due to the formation of hydrogen bonding of the -OH groups of the polymeric matrix with the NPs-SiO_2_. Also, the XRD crystallographic study confirmed the amorphous nature of the PVA/CS mixture, which decreases with amorphous diffraction peaks at 2θ = 19.3 and 22.5 attributed to the 110 and 200 planes. With the addition of NPs-SiO_2_ and the decrease in the CS percentage in F2–F3, the intensity of the peaks decreased, evidencing a loss of crystallinity. On the other hand, the increase in NPs-SiO_2_ produced a strengthening effect and improved the thermal stability. SEM analysis determined that the incorporation of nanoparticles in the polymeric mixtures provides a more compact and homogeneous morphology due to the strong intermolecular hydrogen bonds of PVA, and the reinforcement with nanoparticles allowed a denser molecular packing, acting synergistically, facilitating the dispersion of the components on the polymeric matrix and breaking its narrow intramolecular structure. On the other hand, it was possible to observe the inhibitory effect of the scaffolds.

Silicon dioxide nanoparticles (NPs-SiO_2_) have shown promising antimicrobial properties against various bacteria. This study demonstrates that NPs-SiO_2_ effectively inhibits the growth of Gram-negative bacteria such as *E. coli* and *E. cloacae*. This antimicrobial effect is attributed to the ability of NPs-SiO_2_ to disrupt the bacterial cell wall through electrostatic interactions. However, the efficacy of NPs-SiO_2_ decreases against Gram-positive bacteria such as *S. aureus* due to a thicker peptidoglycan layer in their cell walls, which, together with the hydrophobic character and chelating ability of CS, provide enhancements in the interactions of amino groups and active sites present in the bacterial membrane. These PVA/CS/NPs-SiO_2_ scaffolds demonstrated robust antimicrobial activity against a broad spectrum of bacteria, including *S. aureus*.

Regarding biocompatibility, the PVA/CS/NPs-SiO_2_ scaffolds were compatible and resorbable; the degradation and resorption process in the hydrolytic degradation pathway due to the presence of PVA and the cellular enzymatic pathway via the CS in conjunction with NPs-SiO_2_ attributed a progressive replacement of the implanted material with a maturing connective tissue. After implantation, there was no adverse reaction since the PVA guarantees the fragmentation of the scaffold, and by decreasing the percentage of CS and increasing the percentage of nanoparticles, there was more significant resorption of the material and the need to phagocytose the fragments of the blocks stimulating the proliferation of collagen fibers, blood vessels, and cells. The F4 formulation differs from the other three in that mature connective tissue progressively replaced the fibrous capsule. All the results presented expand the frontiers of tissue engineering and regenerative medicine by highlighting the crucial role of nanoparticles in optimizing scaffold properties.

## Figures and Tables

**Table 1:** Thermal properties of PVA/CS/NPs-SiO_2_ scaffolds.

	T_g_ (°C)	T_m1_ (°C)	T_m2_ (°C)	T_m3_ (°C)
F1	74	84	147	315
F2	-	80	147	315
F3	76	88	147	315
F4	77	84	147	319

**Table 2:** Mechanical properties of PVA/CS/NPs-SiO_2_ scaffolds.

Formulation	M. Elastic (MPa) *	Max. Compressive Stress (MPa) *	Max. Deformation (%) *
F1	0.0364 ± 0.01 ^a^	0.575 ± 0.2 ^bc^	211.8 ± 101.0 ^a^
F2	0.0455 ± 0.02 ^a^	1.028 ± 0.2 ^ab^	171.9 ± 48.0 ^a^
F3	0.0443 ± 0.03 ^a^	1.180 ± 0.4 ^a^	176.2 ± 33.9 ^a^
F4	0.00362 ± 0.02 ^a^	0.3353 ± 0.08 ^c^	243.4 ± 82.3 ^a^

* Different letters in the same column indicate significant differences (*p* < 0.05).

**Table 3:** Minimum inhibition concentration (MIC) of NPs-SiO_2_ (mg/mL).

Microorganism	MIC (mg/mL)
*K. pneumoniae*	25.0 ± 1.0
*E. cloacae*	3.13 ± 0.13
*E. coli*	3.13 ± 0.14
*S. enterica* ATCC 53648	25.0 ± 1.0
*S. aureus* ATCC 55804	n.d *

* n.d.: not determined.

**Table 4:** Evaluation of the antimicrobial activity expressed as percentage inhibition of nanocomposites synthesized using strains of *K. pneumoniae*, *E. cloacae*, *S. enterica* ATCC 53648, and *S. aureus* ATCC 55804.

Strain	F1	F2	F3	F4
*K. pneumoniae*	99.88 ± 0.10 ^a^	100 ± 0.0 ^a^	100 ± 0.0 ^a^	99.97 ± 0.02 ^a^
*E. cloacae*	99.98 ± 0.02 ^a^	99.66 ± 0.23 ^a^	99.79 ± 0.14 ^a^	96.92 ± 2.75 ^a^
*S. enterica* ATCC 53648	67.38 ± 1.13 ^c^	63.5 ± 4.0 ^c^	n.d.	47.75 ± 6.25 ^b^
*S. aureus* ATCC 55804	99.53 ± 0.28 ^b^	99.61 ± 0.11 ^b^	98.55 ± 0.89 ^b^	82.88 ± 11.42 ^a^

Different letters in the same row indicate significant differences (*p* < 0.05). n.d.: not determined.

**Table 5:** Formulations used for scaffolds PVA/CS/NPs-SiO_2_.

Components	F1 (%)	F2 (%)	F3 (%)	F4 (%)
PVA	70	70	70	70
CS	30	29.5	29	28
NPs-SiO_2_	-	0.5	1	2

## Data Availability

Data will be available upon request from the corresponding author.
